# Forest Tree and Woody Plant-Based Biosynthesis of Nanoparticles and Their Applications

**DOI:** 10.3390/nano15110845

**Published:** 2025-06-01

**Authors:** Abubakr M. J. Siam, Rund Abu-Zurayk, Nasreldeen Siam, Rehab M. Abdelkheir, Rida Shibli

**Affiliations:** 1 Department of Horticulture & Crop Science, Faculty of Agriculture, University of Jordan, Amman P.O. Box 11942, Jordan; abmjsiam@gmail.com (A.M.J.S.); r.shibli@ju.edu.jo (R.S.); 2 Department of Forestry & Range Science, Faculty of Environmental Sciences & Natural Resources, University of Al Fashir, El Fasher P.O. Box 125, Sudan; 3 Hamdi Mango Center for Scientific Research, The University of Jordan, Amman P.O. Box 11942, Jordan; 4 Department of Industrial Chemistry, College of Applied & Industrial Science, University of Bahri, Khartoum P.O. Box 1660, Sudan; nasreldeennono11@hotmail.com; 5 Department of Forestry, College of Natural Resources & Environmental Studies, University of Bahri, Khartoum P.O. Box 1660, Sudan; rhab_mhmood@hotmail.com

**Keywords:** nanotechnology, green synthesis, forest tree, plant parts, nanoparticle applications

## Abstract

Forest ecosystems represent a natural repository of biodiversity, bioenergy, food, timber, water, medicine, wildlife shelter, and pollution control. In many countries, forests offer great potential to provide biogenic resources that could be utilized for large-scale biotechnological synthesis and products. The evolving nanotechnology could be an excellent platform for the transformation of forest products into value-added nanoparticles (NPs). It also serves as a tool for commercial production, placing the forest at the heart of conservation and sustainable management strategies. NPs are groups of atoms with a size ranging from 1 to 100 nm. This review analyzes the scholarly articles published over the last 25 years on the forest and woody plant-based green synthesis of NPs, highlighting the plant parts and applications discussed. The biosynthesis of nanomaterials from plant extracts provides inexpensiveness, biocompatibility, biodegradability, and environmental nontoxicity to the resultant NPs. The leaf is the most critical organ in woody plants, and it is widely used in NP biosynthesis, perhaps due to its central functions of bioactive metabolite production and storage. Most biosynthesized NPs from tree species have been used and tested for medical applications. For sustainable advancements in forest-based nanotechnology, broader species coverage, expanded applications, and interdisciplinary collaboration are essential.

## 1. Introduction

**Figure 1:**
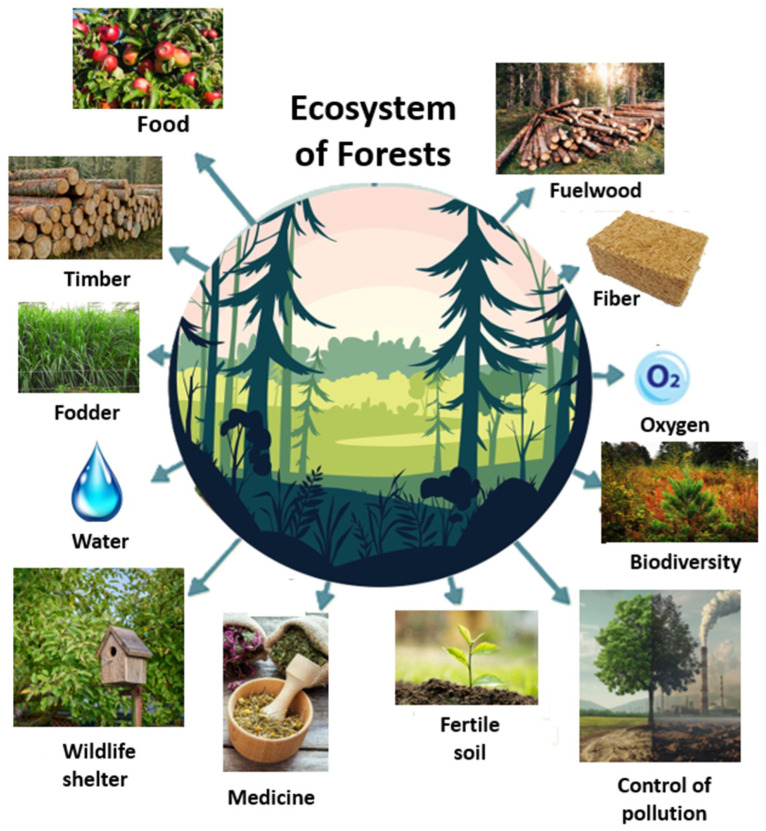
The forest ecosystem as a vital source of resources and environmental services.

Forests are fundamental natural resources that provide essential socioeconomic, environmental, educational, medical, and climate change mitigation services [1,2,3,4,5,6]. The ecosystems of forests represent a natural repository of food, fuelwood, timber, fiber, fodder, oxygen, water, biodiversity, fertile soil, medicine, wildlife shelter, and pollution control (Figure 1).

In many countries, forests offer great potential and could be utilized as an indispensable source for large-scale biotechnological synthesis and economic benefits [7,8,9,10]. Forests contain about 80% of the terrestrial biodiversity of the Earth’s biosphere [11]. With the renowned advancements in nanotechnology in different fields of life, it is possible to broadly exploit forest resources and diversity to produce various nanostructured materials and composites. Nanotechnology is an evolving and growing field in science, with many applications, especially in the biosciences and technology, resulting in new products from nanoparticles [12,13,14,15]. According to Javad et al. (2017) [16], nanoparticles (NPs) are defined as groups of atoms with a size ranging from one to one hundred nanometers (nm). Nanotechnology is a multidisciplinary science that deals with matter at the nanoscale size. Thus, nanomaterials (NMs) can be defined as materials with internal or external structures within the nanoscale dimension [17].

In recent decades, the biosynthetic approach has been widely adopted, using biological organisms such as plant extracts, fungi, and bacteria as viable and simple tools relative to routinely used toxic chemicals [16,18,19,20,21]. The chemical preparation of nanoparticles is the most well-known method, but the biological synthesis of nanomaterials has proven to be much simpler, more inexpensive, and more ecofriendly [16,22,23,24]. The effective use of renewable natural resources for innovative green synthesis is key to sustainable development in green chemistry [25,26]. The use of bio-based nanoparticles has gained particular attention because of their potential in plant production, medical uses, and industrial development, among other applications [27,28,29,30].

## 2. Forest Tree and Woody Plant-Based Synthesis of Nanoparticles

**Figure 2:**
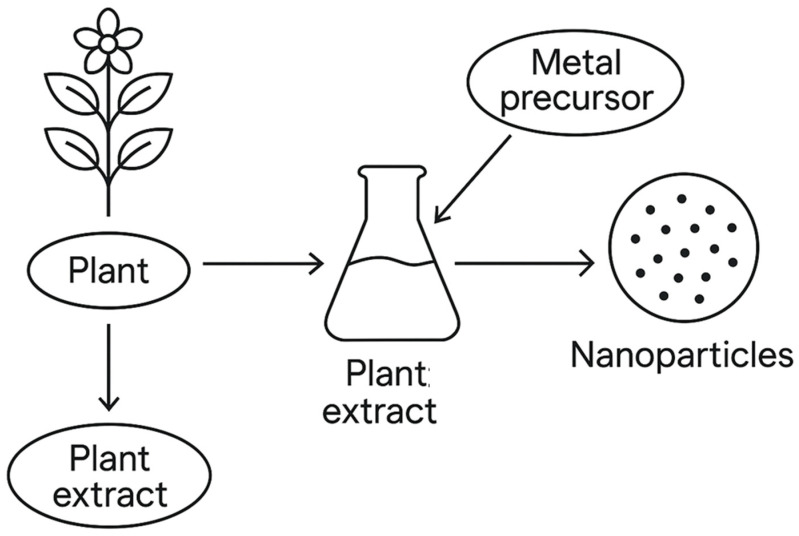
Biosynthesis of nanoparticles.

The green synthesis of nanoparticles from plant extracts is receiving notable interest due to their natural abundance, broad spectrum of bioactive metabolites, and vast applications in industrial, medical, agricultural, and environmental aspects [31,32,33,34]. Various plant parts are used for the synthesis of nanomaterials as they contain numerous compounds, such as steroids, gums, resins, polyphenols, saponins, terpenoids, alkaloids, fibers, rubber, latex, antioxidant enzymes, and others, which act as reducing and stabilizing agents in nanoparticle synthesis [17,19,35,36]. It has been reported that the use of plant materials as bio-reducing agents for the synthesis of nanoparticles is a cost-effective and environmentally benign option compared to the conventional chemical approach [26,37,38]. The green synthesis of nanoparticles using plant-biobased materials is shown in Figure 2. Forest trees and woody plants represent fundamental biogenic resources for the synthesis of NPs, which can also be used for further improvements in forestry, agricultural crops, medicine, and industrial sectors [39,40,41].

The biosynthesis of nanomaterials from woody plant extracts provides biocompatibility, biodegradability, and environmental nontoxicity to the resultant nanoparticles [16,42,43,44,45,46,47]. Furthermore, biogenic material synthesis tends to be a more economical, simpler, and robust route for the synthesis of nanoparticles [48,49]. Green synthesis is a component of biotechnology that entails using the expertise and techniques of biology to manipulate molecular, genetic, and cellular processes, serving to increase the resulting services and products for utilization in numerous fields, including medicine, agriculture, forestry, and environmental safety [50,51]. In the forestry sector, biotechnology is considered to be an excellent platform for the transformation of forest materials into value-added products and serves as a tool for commercial production, placing the forest at the heart of conservation and sustainable management strategies [9,17]. Although forests tend to be fundamental biogenic sources for nanomaterial synthesis and an essential application hub, there are currently limited reviews in the forestry domain [51,52].

This review paper evaluates and analyzes the information published over the past 25 years regarding the green synthesis of nanoparticles (NPs) from forest and woody plant species and their components. Before delving into the analysis, we summarize the key parameters influencing the nanoparticles synthesized from plant extracts.

The green synthesis of nanoparticles using forest plant extracts relies heavily on several critical parameters that impact the efficiency, morphology, and stability of the resulting nanomaterials. One of the most significant factors is the pH; alkaline conditions accelerate the reduction of metal ions, typically leading to smaller and more stable nanoparticles, while acidic conditions slow the reduction process, often resulting in larger particles or aggregates [20,23]. The temperature is another vital variable—higher synthesis temperatures can speed up the reduction and nucleation rates, often yielding smaller nanoparticles. However, excessively high temperatures may degrade the plant-derived biomolecules that act as capping and stabilizing agents, thereby diminishing the nanoparticles’ stability [31]. The concentration of the plant extract is also crucial, as the bioactive compounds within the extract reduce metal ions and stabilize the nanoparticles, necessitating an optimal concentration to balance nucleation and growth. Excessive concentrations may lead to particle agglomeration due to an overload of organic compounds [23,31]. Likewise, optimizing the concentration of the metal salt is essential; lower concentrations promote the formation of nanoparticles within the nanoscale range, while higher concentrations can cause uncontrolled nucleation and aggregation [31]. Furthermore, the reaction time and stirring significantly affect the synthesis process. Extended reaction times can improve the stability and uniformity of nanoparticles but may also lead to larger particle sizes. Stirring facilitates even interaction between metal ions and phytochemicals, enhancing the consistency of the particle morphology [32]. Finally, the method employed to extract phytochemicals from plant materials—such as boiling, cold maceration, or sonication—can affect both the type and amount of bioactive compounds, ultimately influencing the nanoparticles’ characteristics, such as their size, shape, and functional properties [33].

### 2.1. Biosynthesis of NPs Using Leaf Extracts

Leaf extracts from many tree species are used to prepare a broad range of metal and metal oxide NPs, as shown in Table 1. The leaves of *Eucalyptus, Mimusops*, *Azadirachta*, *Ekebergia*, *Abies*, *Debregeasia*, *Dalbergia*, and various other tree species are used in the synthesis of silver NPs [40,53,54,55,56,57]. Silver chloride NPs are also produced using the leaves of kaffir lime (*Citrus hystrix*) [58], and green zero-valent iron NPs are produced using oak (*Quercus* spp.), mulberry (*Morus alba*), and cherry (*Prunus cerasus*) tree leaves [59]. Gold and iron NPs are prepared using leaf extracts from *Acer pentapomicum*, *Ficus benghalensis*, and *Albizia lebbeck* [60,61,62].

Various oxide NPs, including CuO NPs, MgO NPs, ZnO NPs, Fe_2_O_3_ NPs, and others, are fabricated from the leaf extracts of woody tree species [47,63,64,65,66]. It is noted that the leaf is regarded as the most important part of forest and woody plants used in NP biosynthesis, perhaps due to its central functions of metabolic activity and metabolite storage. Biologically active metabolites, such as terpenes, phenols, glycosides, alkaloids, and others, with antimicrobial activity, detergent properties, and pharmacological applications, are mainly produced in plant leaves and will be relocated to other parts when required [67,68,69,70].

**Table 1:** Biosynthesis, size characterization, and applications of nanoparticles (NPs) using leaf extracts from various tree species.

Tree Species/Part Used	Tree Family	Chemical Substrate Used for NP Synthesis	Nanoparticles (NPs)/ Size of NPs	Applications of NPs	Reference
*Eucalyptus camaldulensis*/leaves	*Myrtaceae*	Silver nitrate (AgNO_3_) solution	Silver nanoparticles (Ag NPs) 8.7–26.5 nm	enhancement of germination rate and seedling growth	[41]
*Eucalyptus camaldulensis*/leaves	*Myrtaceae*	AgNO_3_	Ag NPs ˂100 nm	showed antibacterial activity	[71]
*Eucalyptus chapmaniana*/leaves	*Myrtaceae*	AgNO_3_	Ag NPs 60 nm	inhibited six pathogenic organisms’ activity and reduced viability of HL-60 cells in a dose-dependent manner	[72]
*Ecalyptus camaldulensis*/leaves	*Myrtaceae*	AgNO_3_	Ag NPs 28–68 nm	not included	[56]
*Eucalyptus grandis*/leaves	*Myrtaceae*	AgNO_3_	Ag NPs 1.7–52.9 nm	presented antibacterial activity	[73]
*Eucalyptus oleosa*/leaves	*Myrtaceae*	AgNO_3_	Ag NPs 21 nm	not included	[74]
*Mimusops elengi*/leaves	*Sapotaceae*	AgNO_3_	Ag NPs 4–28 nm	not included	[54]
*Azadirachta indica*/leaves	*Meliaceae*	AgNO_3_	Ag NPs 10–100 nm	not included	[53]
*Ekebergia capensis*/leaves	*Meliaceae*	AgNO_3_	Ag NPs 20–120 nm	antimicrobial effects against oral biofilm-forming bacteria	[55]
*Abies webbiana*/leaf	*Pinaceae*	AgNO_3_	AW-Ag NPs Not included	showed antibacterial activity	[57]
*Debregeasia salicifolia*/leaves	*Urticaceae*	AgNO_3_	Ag NPs 38.15 nm	increased antibacterial activity	[75]
*Carissa carandas*/leaves	*Apocynaceae*	AgNO_3_	Ag NPs 28–06 nm	exhibited antioxidant, anticancer, and antibacterial properties	[76]
*Thevetia peruviana*/leaves	*Apocynaceae*	AgNO_3_	Ag NPs 6.4–39.4 nm	showed activity against fungal pathogens and bacteria	[77]
*Dalbergia sissoo*/leaf	*Fabaceae*	AgNO_3_	Ag NPs 10–25 nm	showed significant capacities against harmful bacteria	[40]
*Mimusops elengi*/leaf	*Sapotaceae*	AgNO_3_	Ag NPs 55–83	showed higher antimicrobial efficacy against multidrug-resistant clinical isolates	[78]
*Prosopis juliflora*/leaf	*Mimosaceae*	AgNO_3_	Ag NPs 10–20 nm	degraded toxic compounds in a short time	[79]
*Ziziphus Jujuba*/leaf	*Rhamnaceae*	AgNO_3_	Ag NPs 20–30 nm	reduced environmental pollution effects of 4-nitrophenol (4-NP) and Methylene Blue, exhibited antimicrobial activity against *Escherichia coli*	[80]
*Acer pentapomicum*/leaf	*Sapindaceae*	Gold chloride	Au NPs 19–24 nm	exhibited good antioxidant, antibacterial, and antifungal activity	[62]
*Albizia lebbeck*/leaves	*Mimosaceae*	Fecl_3_·6H_2_O	Iron nanoparticles ,size not included	able to remove Crystal Violet and Congo Red dyes	[61]
*Diospyros montana*/leaf extract	*Ebenaceae*	AgNO_3_	Ag NPs 61.69 nm	exhibited antibacterial effects against nine pathogenic bacterial strains	[81]
*Ficus benghalensis*/leaf extract	*Moraceae*	AgNO_3_	Ag NPs 16 nm	showed effective antibacterial activity toward *E.coli*	[82]
*Ficus benghalensis*/leaf extract	*Moraceae*	Gold chloride	Au NPs 2–100 nm	demonstrated potent antimicrobial activity against some Gram-positive and -negative bacteria	[60]
*Juglans regia*/leaf extract	*Juglandaceae*	AgNO_3_	Ag NPs 10–50 nm	not included	[83]
*Citrus hystrix* (kaffir lime)/leaves	*Rutaceae*	Silver nitrate and gelatin	Ag and AgCl nanoparticles 20–50 nm	exhibited antibacterial and antiproliferative activity against carcinoma; exhibited negative effect toward human fibroblasts	[58]
*Prosopis juliflora*/leaf extract	*Mimosaceae*	AgNO_3_	Ag NPs 35–60 nm	showed antimicrobial activity in sewage system	[84]
Oak *(Quercus brantii)*/leaves	*Fagaceae*	AgNO_3_	Ag NPs 6 nm	not included	[85]
Teak (*Tectona grandis*)/leaves	*Verbenaceae*	AgNO_3_	Ag NPs 26–28 nm	demonstrated antibacterial activity against pathogenic bacteria such as *S. aureus* and *E. coli*	[86]
*Acacia nilotica*/leaves	*Mimosaceae*	AgNO_3_	Ag NPs 20 nm	exhibited antineoplastic efficacy toward SKOV3 ovarian cancer cells	[87]
*Cestrum nocturnum*/leaf extract	*Solanaceae*	AgNO_3_	Ag NPs 20 nm	had strong antioxidant and antibacterial activity due to the presence of bioactive molecules on the surfaces of NPs	[36]
*Dalbergia sissoo*/leaf extract	*Fabaceae*	Mg(NO_3_)_2_·6H_2_O	MgO NPs ˂50 nm	degraded Methylene Blue dye, exhibited antibacterial effects against *Escherichia coli* and *Ralstonia solanacearum* strains	[47]
*Melia azedarach*/leaf extract	*Meliaceae*	Zn(NO_3_)_2_6H_2_O	MaZnO NPs 30–40 nm	prevented the growth of seed-borne fungal pathogens in soybean	[66]
*Agrewia optiva* and *Prunus persica*/leaf extracts	*Malvaceae* and *Rosaceae*	Iron(II) chloride tetrahydrate (FeCl_2_·4H_2_O)	Iron oxide nanoparticles 14–17 nm	exhibited effective antibacterial activity against Gram-positive and Gram-negative bacteria	[64]
*Albizia lebbeck*/leaf extract	*Mimosaceae*	Copper sulfate	Copper oxide nanoparticles (CuO NPs) ˂100 nm	not included	[63]
*Tectona grandis*/leaf extract	*Verbenaceae*	Zinc nitrate	Zinc oxide nanoparticles (ZnO NPs) 54 nm	exhibited antibacterial activity against Gram-positive and Gram-negative bacteria and anticancer effects	[88]
*Terminalia catappa*/leaf	*Combretaceae*	Nd(NO_3_)_3_	Neodimium oxide nanoparticles (Nd_2_O_3_ NPs) 40–60 nm	not included	[65]
*Rhus punjabensis*/leaf extract	*Anacardiaceae*	Ferric chloride	Iron oxide NPs 41.5 nm	showed free radical scavenging and cytotoxic potential against cancer cell lines	[89]
*Rosa brunonii*/leaves	*Rosaceae*	AgNO_3_	Ag NPs ˂100 nm	showed antimicrobial activity against different microbial strains and degradation of Congo Red dye	[89]
Oak (*Quercus peatrea*), mulberry (*Morus alba*), and cherry (*Prunus cerasus*)/leaves	*Fagaceae,* *Moraceae*, and *Rosaceae*	Fe(III) solution	Green zero-valent iron nanoparticles (nZVIs) 10–30 nm	showed potential for use in remediation of water matrices contaminated with As(III) and Cr(VI)	[59]

### 2.2. Biosynthesis of NPs Using Fruit and Seed Extracts

A wide range of extracts from the fruits and seeds of woody plant species are used to manufacture metal, metal oxide, and quantum dot NPs (Table 2). The pods of *Acaia nilotica* and *Parkia speciosa* tree species were used to biosynthesize silver, iron, and tin oxide quantum dot nanoparticles [45,90,91,92]. Silver NPs were prepared using the fruit of *Ficus benghalensis* [93], the fruit pericarp of *Sapindus emarginatus* [94], the fruit peel of *Citrus* species [95], the fruit hulls of *Quercus* species [96], the cones of pine tree species [97], and seed extracts from teak [98] and other tree species. Fruit bark extracts from the oak tree are used in the synthesis of palladium NPs [99], and the walnut *(Juglans regia)* tree fruit husk has been used to fabricate silver chloride NPs [100]. The fruit hull extract of the oak tree was also used in producing ZnO and CuO NPs [101], as well as bimetallic silver/zinc oxide NPs [44].

### 2.3. Biosynthesis of NPs Using Stem Bark Extracts

The biosynthesis of nanomaterials using stem bark extracts from woody plant species is an increasing trend (Table 3). Bark extracts from numerous tree species from different tree families are used to fabricate metal NPs, particularly silver NPs [109,110]. Silver NPs have been produced using the bark extracts of tree species from the families *Mimosaceae* [7,46,111,112], *Moraceae* [110,113], and *Combretaceae* [42,114], as well as tree species such as *Boswellia ovalifoliolata* and *Pinus eldarica* [115,116]. As populations in many regions, such as Africa, South America, and East Asia, are still using raw stem bark in the treatment of various ailments, scientific investigations should continue to elucidate the types and amounts of bioactive metabolites in the bark of various tree species [117,118,119,120].

### 2.4. Biosynthesis of NPs Using Extracts of Different Plant Parts and Constituents

Various review articles have described the green synthesis of NPs using different parts and exudates from woody plants, such as gums, latex, rubber, oils, pollens, roots, cellulose, and mixed extracts from the roots and stems (Table 4). The rubber of *Hevea brasiliensis,* gum of *Anogeissus latifolia,* and latex of *Ficus sycomorus* and *Calotropis procera* were used to produce silver NPs [122,123,124,125]. Silver NPs were also fabricated using pollens from pine [126], roots from palm [127], and extracts from an *Acacia rigidula* root stem mixture [128]. Furthermore, oil of bitter orange (*Citrus aurantium*), leaf cellulose from screw pine (*Pandanus tectorius*), stem fiber from rubberwood (*Hevea brasiliensis*), and the tree and fruit pulp from oil palm (*Elaeis guineensis*) were used in the biosynthesis of chitosan NPs, cellulose nanocrystals, and cellulose nanofibers, respectively [129,130,131]. This broad range of extracts and constituents gained from various parts of woody plants, with potential suitability for NP fabrication, offers a promising and sustainable basis for biogenic resource-based nanotechnology if it is managed well.

## 3. Applications of Woody Plant-Based Biosynthesized NPs 

As shown in Table 1, Table 2, Table 3 and Table 4, applications of woody plant-based biosynthesized NPs as nanofertilizers and stimulators for plant growth have been documented. It has been reported that Ag NPs can be used to promote *Acacia senegal* and *Acacia mellifera* growth [41], and Fe_2_O_3_ NPs in *Photinia fraserii* and *Cotinus coggygria* promoted tree seedling growth [132]. Zinc oxide nanoparticles extracted from *Citrus limon* and *Melia azedarach* tree leaves were applied as pesticides for the protection of sweet potato and soybean, respectively, against pathogens [66,105], and chitosan NPs derived from *Citrus aurantium* were used to preserve the postharvest quality of button mushrooms [131]. According to the available literature, most biosynthesized nanoparticles from tree species have been used and tested for medical and antimicrobial applications. Silver NPs extracted from many tree species and families show biomedical effects [78,79] and antibacterial [40,55,95,121], antifungal [77,126], antioxidant [62,76], anticancer [107,111], and toxic degradation properties [79,80]. Iron and copper oxide nanoparticles obtained from *Acacia nilotica* exhibited an ability to eliminate several pathogens from human samples [91,104]. Gold NPs from *Ficus benghalensis* [60], iron oxide NPs from *Agrewia optiva* and *Prunus persica* [64], and zinc oxide NPs extracted from teak trees [88] have demonstrated potent antimicrobial activity against some Gram-positive and -negative bacteria.

It is worth mentioning that green synthesis may have negative consequences for the environment and plant habitats as the processes imply modifications in cellular, genetic, and molecular aspects. The nanoparticles’ quality, stability, size, and shape vary from one tree species to another, and the phytochemical richness significantly differs across plant species [17,133]. Therefore, the assessment of green synthesis and the applications of nanoparticles, their benefits, and their drawbacks must be performed concurrently. For sustainable research on nanotechnology in forestry, the coverage of a large range of tree species, alongside adopting an interdisciplinary collaboration approach that involves different institutions worldwide, would be of prime interest.

## 4. Comparative Overview

The analysis of the studies indicates that the leaves are the most frequently utilized plant parts for nanoparticle synthesis, comprising roughly 47% of all cases identified in our review. This preference likely stems from the high concentrations of bioactive phytochemicals in the leaves, which promote effective nanoparticle formation. Following the leaves, the fruits, seeds, and related structures (such as the fruit pericarp, testa, husk, hull, and cones) account for approximately 21% of the total, while the bark contributes 17%. Other plant parts, like the stems, roots, latex/gum/oil, and pollen, are used less often, together representing the remaining proportion.

This strong preference for leaves arises from several factors. Leaves are typically more accessible and renewable than other plant parts, which enables sustainable harvesting without causing significant harm to the plant. More importantly, they are abundant in a variety of bioactive phytochemicals, such as flavonoids, phenolics, and tannins, which act as effective reducing and stabilizing agents in the green synthesis of nanoparticles. Additionally, the large surface area and high metabolic activity of the leaves further improve the extraction of these compounds, leading to more efficient and reproducible nanoparticle formation.

Ag NPs represent the most widely synthesized nanoparticles, comprising 75% of the syntheses exhibited in this review. This dominance is primarily due to their well-established broad-spectrum antimicrobial properties, which make them highly sought after for a range of biomedical, environmental, and industrial uses. Silver ions are notably effective against numerous pathogenic microorganisms, with their antimicrobial effectiveness significantly heightened when reduced to the nanoscale, thanks to their increased surface areas and reactivity. Additionally, the synthesis of Ag NPs using plant extracts is an environmentally friendly process that is both straightforward and efficient. Plant phytochemicals like flavonoids, phenolics, tannins, and terpenoids function as reducing and stabilizing agents, facilitating the rapid and eco-conscious conversion of silver ions into stable nanoparticles. The interplay between silver chemistry and plant biochemistry allows a wide variety of plant species and parts to be used for Ag NP production, usually under mild reaction conditions and without requiring toxic chemicals or high-energy inputs. Moreover, the observable color change during Ag NP formation provides a simple, immediate sign of successful synthesis, enhancing their appeal in research.

Other nanoparticles, such as zinc oxide, gold, iron, copper oxide, and others, make up the remaining 25%. While these alternative nanoparticles showcase valuable properties, like photocatalytic activity, antioxidant effects, and targeted therapeutic potential, their synthesis using plant extracts is often more complex or less efficient. Additionally, despite their growing ranges of applications, they do not enjoy the same widespread recognition as Ag NPs. Consequently, Ag NPs continue to be the primary focus of research in plant-mediated nanoparticles, thanks to their proven effectiveness, straightforward synthesis, and versatility across diverse scientific and practical domains.

In terms of application, plant-derived nanoparticles are predominantly used for their antimicrobial properties, making up about 69% of the reported applications. This significant emphasis highlights the large global demand for innovative strategies to address microbial pathogens, particularly in light of the alarming increase in antibiotic-resistant bacteria and the drawbacks of traditional antimicrobial agents. Silver nanoparticles (Ag NPs), in particular, demonstrate broad-spectrum effectiveness against various bacteria, including both Gram-positive and Gram-negative types, as well as some fungi and viruses. Their potent antimicrobial activity stems from their capacity to disrupt microbial cell membranes, produce reactive oxygen species, and affect critical cellular functions, rendering them highly effective even against drug-resistant strains. The preference for antimicrobial research is also influenced by the straightforward and clear testing methods available, along with the direct and quantifiable results that they yield. Additionally, employing plant extracts for nanoparticle synthesis creates distinctive phytochemical coatings on the nanoparticles’ surfaces, enhancing their biocompatibility and further elevating their antimicrobial efficacy. This interaction between plant-derived substances and metallic nanoparticles significantly contributes to the prevalence of antimicrobial applications in the field.

Other significant applications include antioxidants (9%), anticancer/antiproliferative agents (13%), and environmental remediation or catalytic functions, like dye degradation (14%). Antioxidant uses harness the radical-scavenging properties of both nanoparticles and their phytochemical capping agents, offering potential advantages in food preservation, cosmetics, and medicine. The use of plant-based nanoparticles in anticancer and antiproliferative contexts is on the rise, with multiple studies showing that these nanoparticles can trigger apoptosis or slow down the growth of various cancer cell lines, typically with lower toxicity to healthy cells compared to traditional chemotherapeutics. Environmental remediation and catalytic functions, including the degradation of harmful dyes and pollutants, highlight the promise of these nanomaterials for sustainable water treatment and pollution management. Although antifungal and other specialized applications are reported less frequently, they are becoming increasingly noteworthy as the adaptability of plant-based nanoparticles gains recognition.

These trends collectively underscore the crucial role of leaf-derived phytochemicals in the eco-friendly synthesis of silver nanoparticles, which are primarily studied for their potent antimicrobial properties. At the same time, the data show a growing, albeit still secondary, interest in expanding the use of plant-based nanomaterials to address broader challenges in medicine, environmental remediation, and industry. This trend reflects both the proven efficacy and adaptability of Ag NPs and ongoing research into new plant sources, types of nanoparticles, and novel applications to meet future technological and societal demands.

## 5. Current Challenges and Research Opportunities in Forest Tree-Based Nanoparticle Synthesis

The synthesis of forest tree-based nanoparticles provides an eco-friendly alternative to traditional methods. Nevertheless, various challenges limit its broader use.

- The biochemical composition of tree extracts can vary due to the species, age, and environmental conditions, significantly impacting the nanoparticle synthesis outcomes. This variability influences the nanoparticle size, shape, and stability. For example, a study comparing silver nanoparticle synthesis with the use of extracts from collard greens, hazelnut, and green tea revealed that green tea extract, abundant in phenolic compounds, accelerated nanoparticle formation and produced smaller, more stable particles. In contrast, collard greens, having lower phenolic content, resulted in less effective nanoparticle synthesis with larger, more polydisperse particles [134]. Additionally, seasonal changes can modify plants’ phytochemical profiles [135].
- The lack of standardized protocols for plant-mediated nanoparticle synthesis creates considerable challenges regarding reproducibility and scalability. Differences in the plant species, cultivation conditions, and extraction methods may lead to inconsistencies in properties such as the size, shape, and stability of nanoparticles. For instance, variability in the phytochemical content of extracts, influenced by environmental and species factors, can cause batch-to-batch discrepancies in the synthesis results. This highlights the urgent need to standardize the extraction and synthesis methods to achieve consistent and reproducible outcomes across various studies and applications.
- The mass production and disposal of nanoparticles present serious environmental hazards, mainly because their release into air, water, and soil can result in ecosystem contamination and toxicity. Manufactured nanomaterials can enter the environment through intentional and accidental means, including emissions from production sites, waste discharge, spills during transport, and the degradation of consumer items [136].
- This necessitates detailed impact assessments. Research indicates that NPs can trigger oxidative stress, DNA damage, and inflammation in various organisms, threatening human health and ecosystems [137]. Furthermore, releasing nanoparticles into the environment might result in their accumulation in soil and water, potentially disrupting microbial communities and nutrient cycles [138]. Thus, a thorough evaluation of the environmental implications of nanoparticle synthesis and their applications is crucial, ensuring the adoption of sustainable practices to mitigate the adverse effects.
- Despite these obstacles, there are encouraging research prospects.
- The phytochemical profiling of tree extracts is vital in pinpointing the key compounds involved in NP synthesis, facilitating more controlled and efficient processes. Research has shown that various bioactive compounds in plant extracts, including polyphenols, flavonoids, terpenoids, and alkaloids, can serve as reducing and stabilizing agents during NP formation [139]. This highlights the significance of comprehensive phytochemical analysis in uncovering the nanoparticle synthesis mechanisms and optimizing the processes for targeted results.
- Utilizing forest waste materials such as sawdust, bark, and leaves for nanoparticle synthesis provides a sustainable approach that diminishes the environmental impact while also enhancing the value of by-products usually seen as waste. For example, lignin nanoparticles (LNPs) have been effectively extracted from the sawdust of Iroko (*Milicia excelsa*) and Norway spruce (*Picea abies*) trees. These LNPs were subsequently used to coat beech (*Fagus sylvatica*) wood, increasing its resistance to artificial weathering, showcasing wood waste’s potential in generating functional nanomaterials [140]. This emphasizes the practicality of using forest waste materials in nanoparticle synthesis, encouraging sustainability and reducing waste in the forest product industry.
- Environmental impact studies play a critical role in shaping the development of eco-friendly strategies for nanoparticle synthesis. Life cycle assessments (LCAs) have been utilized to evaluate the environmental effects of green synthesis methods versus traditional ones, showcasing advantages like decreased emissions and energy use. For example, an LCA focused on iron oxide nanoparticle synthesis using natural extracts highlighted a more sustainable approach with lower environmental burdens [141]. These evaluations reinforce the necessity of implementing green synthesis techniques to lessen ecological footprints and advocate for sustainability in the production of nanomaterials.

## 6. Conclusions

A review of the existing literature on the green synthesis of nanoparticles indicates that various forest and woody plants from diverse species and families serve as biogenic sources. The significant advancements in nanotechnology can be applied to harness the extensive resources and biodiversity found in forests to create a variety of nanostructured materials and composites. Green biosynthesis provides an environmentally friendly, cost-effective, and biocompatible method of creating nanoparticles, especially when compared to traditional chemical methods. The application of bio-based nanoparticles has garnered particular interest due to their potential roles in plant enhancement, medical applications, and industrial growth, among others. In the forestry sector, biotechnology is recognized as a valuable avenue for the transformation of forest products into nanoparticles with added value, serving as a means for commercial production and conservation initiatives. A review of the current academic literature shows that the most commonly biosynthesized nanoparticles from tree species have been explored for their medical and antimicrobial properties. However, adverse effects, particularly those of high concentrations of nanoparticles on environmental safety and plant health, have also been documented. Further research into nanotechnology’s applications is necessary to thoroughly evaluate the potential benefits and drawbacks related to various forestry aspects. Fostering sustainable nanotechnology research in forestry, including a broad range of tree species and families, is crucial, as is establishing interdisciplinary collaborations among scientific and development organizations. The variability in the phytochemical compositions among tree species and the lack of standardized protocols for the preparation of extracts and the synthesis of nanoparticles can impede the reproducibility of results. Additionally, the environmental implications of nanoparticle production and disposal raise significant concern, highlighting the need for thorough assessments to encourage eco-friendly practices. Opportunities exist to improve phytochemical profiling to pinpoint key compounds for nanoparticle synthesis, use forest waste, and employ advanced characterization methods to better understand nanoparticles’ properties and interactions. These efforts could enhance sustainable practices in nanotechnology within the forestry sector while mitigating the environmental impact risks.

## Figures and Tables

**Table 2:** Biosynthesis, size characterization, and applications of nanoparticles (NPs) using fruit and seed extracts from various tree species.

Tree Species/Part Used	Tree Family	Main Chemical Substrate Used for Nanomaterial Synthesis	Nanoparticles (NPs)/ Size of NPs	Applications of NPs	Reference
*Acacia nilotica*/pods	*Mimosaceae*	Silver nitrate (AgNO_3_) solution	Silver nanoparticles (Ag NPs) ˂100 nm	inhibited various pathogenic microorganisms, reduced viability of human leukemia cell line	[92]
*Ficus benghalensis*/fruit	*Moraceae*	AgNO_3_	Ag NPs 70–90 nm	acted as effective antimicrobial agents against some pathogenic microorganisms	[93]
*Sapindus emarginatus*/pericarp	*Sapindaceae*	AgNO_3_	Silver nanoparticles 5–20 nm	showed remarkable antibacterial activity	[94]
*Acacia nilotica*/pod	*Mimosaceae*	AgNO_3_	Ag NPs 20–30 nm	showed higher electrocatalytic activity in the reduction of benzyl chloride	[102]
*Acacia nilotica*/pods	*Mimosaceae*	Ferrous sulfate (FeSO_4_·7H_2_O), Methyl Orange (MO), and NaOH	Iron nanoparticles (Fe NPs) 39 nm	have potential to mitigate the pathogenicity of several human pathogens	[91]
*Parkia speciosa*/pod extract	*Mimosaceae*	SnCl_4_·5H_2_O	SnO_2_ quantum dots 1.9 nm	exhibited excellent photocatalytic performance with effective degradation activity	[45]
*Citrus limon*, *Citrus sinensis*, and *Citrus tangerina*/fruit peel	*Rutaceae*	AgNO_3_	Ag NPs 10–70 nm for *C. limon*, 5–80 nm for *C. tangerina*, and 10–50 nm for *C. sinensis*	showed antibacterial activity against *Escherichia coli* and *Staphylococcus aureus*	[95]
Oak (*Quercus* spp.)/ fruit bark extract	*Fagaceae*	AgNO_3_	Ag NPs 20–25 nm	showed greater antimicrobial activity toward Gram-positive bacteria	[103]
Oak (*Quercus* spp.)/ fruit bark extract	*Fagaceae*	PdCl_2_	Palladium nanoparticles (Pd NPs) 5–7 nm	not included	[99]
Oak (*Quercus* spp.)/ fruit hull	*Fagaceae*	AgNO_3_	Ag NPs 40 nm	shown as potential agent for human breast cancer therapy	[96]
*Acacia nilotica*/pods	*Mimosaceae*	Copper nitrate	Copper oxide nanoparticles (CuO NPs) 1–100 nm	exhibited antioxidant, anticancer, and thrombolytic activity	[104]
Lemon (*Citrus limon*)/fruit	*Rutaceae*	Not included	ZnO NPs 60.8 nm and TiO_2_ NPs 41.5 nm	inhibited growth and pathogenic activity of *Dickeya dadantii* bacteria in sweet potato	[105]
Oak (*Quercus* spp.)/ fruit hull	*Fagaceae*	Zinc acetate dihydrate and copper(II) acetate monohydrate	ZnO and CuO nanoparticles 34 nm	exhibited photocatalytic activity for degradation of Basic Violet 3 dye	[101]
Oak (*Quercus* spp.)/ fruit hull	*Fagaceae*	AgNO_3_ and Zn(CH_3_COO)_2_·2H_2_O	Ag NPs, ZnO NPs, and bimetallic silver/zinc oxide nanoparticles (Ag/ZnO NPs) 57 nm for Ag NPs, 34 nm for ZnO, and 19.2 for Ag/ZnO	not included	[44]
Pine (*Pinus* spp.)/ cone extract	*Pinaceae*	AgNO_3_	Ag NPs	exhibited antibacterial action on both Gram classes of bacteria associated with agriculture	[97]
Walnut (*Juglans regia*)/ fruit husk extract	*Juglandaceae*	AgNO_3_	Silver chloride nanoparticles (AgCl NPs) 4–30 nm	showed significant inhibitory effects against *Escherichia coli* and *Staphylococcus aureus* bacteria	[100]
*Annona squamosa*/seeds	*Annonaceae*	AgNO_3_	Ag NPs 20–57 nm	suppressed the growth of pathogenic bacteria	[106]
*Anacardium occidentale*/seed testa	*Anacardiaceae*	AgNO_3_	Ag NPs 25 nm	degraded carcinogenic azo dyes (Congo Red and Methyl Orange)	[107]
Teak (*Tectona grandis*)/seeds	*Verbenaceae*	AgNO_3_	Ag NPs 10–30 nm	showed antimicrobial activity against *Bacillus cereus, Staphylococcus aureus*, and *Escherichia coli* pathogens	[98]
Walnut (*Juglans regia* L.)/ seed extract	*Juglandaceae*	AgNO_3_	Ag NPs 80–90 nm	played important role in photocatalytic degradation of industrial dye effluents	[108]

**Table 3:** Biosynthesis, size characterization, and applications of nanoparticles (NPs) using stem bark extracts from various tree species.

Tree Species/Part Used	Tree Family	Main Chemical Substrate Used for Nanomaterial Synthesis	Nanoparticles (NPs)/ Size of NPs	Applications of NPs	Reference
*Boswellia ovalifoliolata*/stem bark	*Burseraceae*	Silver nitrate (AgNO_3_) solution	Silver nanoparticles (Ag NPs) 30–40 nm	capable of eradicating pathogenic resistant bacteria in an infection in vivo	[115]
*Acacia nilotica*/stem	*Mimosaceae*	AgNO_3_	Ag NPs 2–43 nm	served as potential antimicrobial agent	[112]
*Ficus sycomorus*/stem bark	*Moraceae*	AgNO_3_	Ag NPs 30–75 nm	potent against bacteria (*Shigella*)	[110]
*Shorea tumbuggaia*/stem bark	*Dipterocarpaceae*	AgNO_3_	Ag NPs 40 nm	not included	[109]
*Amentotaxus assamica*/bark	*Taxaceae*	AgNO_3_	Ag NPs 100 nm	showed inhibitory effects against *Escherichia coli* and *Staphylococcus aureus* bacteria	[121]
*Acacia nilotica*/bark	*Mimosaceae*	AgNO_3_	Ag NPs 20–50 nm	possessed versatile biomedical applications	[46]
*Dillenia indica*/bark	*Dilleniaceae*	AgNO_3_	Ag NPs 15–35 nm	showed enhanced free radical scavenging and excellent catalytic activity against toxic chemicals	[43]
*Prosopis juliflora*/*bark*	*Mimosaceae*	AgNO_3_	Ag NPs 10–50 nm	acted as antimicrobial, anticancer, and catalytic agent	[111]
*Terminalia arjuna*/bark	*Combretaceae*	AgNO_3_	Ag NPs 30–50 nm	exhibited significant antibacterial properties	[114]
*Terminalia cuneata*/bark	*Combretaceae*	AgNO_3_	Ag NPs 25–50 nm	showed efficacy in reduction of Direct Yellow 12	[42]
*Acacia leucophloea*/bark	*Mimosaceae*	AgNO_3_	Ag NPs 17–29 nm	exhibited antibacterial activity against common bacterial pathogens	[7]
*Ficus benghalensis* and *Azadirachta indica*/bark	*Moraceae* and *Meliaceae*	AgNO_3_	Ag NPs 85.95 nm for *F. benghalensis* and 90.13 nm for *A. indica*	showed promising antimicrobial activity against Gram-negative and -positive bacteria and showed antiproliferative response against osteosarcoma	[113]
*Pinus eldarica*/bark extract	*Pinaceae*	AgNO_3_	Ag NPs 10–40 nm	not included	[116]

**Table 4:** Biosynthesis, size characterization, and applications of nanoparticles (NPs) using extracts and constituents of various parts of different tree species.

Tree Species/Part Used	Tree Family	Chemical Substrate Used for NP Synthesis	Nanoparticles (NPs)/ Size of NPs	Applications of NPs	Reference
Gum ghatti *(Anogeissus latifolia)*/gum	*Combretaceae*	Silver nitrate (AgNO_3_) solution	Silver nanoparticles (Ag NPs) 5.7 nm	showed significant antibacterial action in both Gram classes of bacteria	[123]
*Ficus sycomorus*/latex and leaf	*Moraceae*	AgNO_3_	Ag NPs ≤20 nm for leaves and ≤100 nm for latex	showed improved antibacterial activity	[125]
*Calotropis procera*/serum latex	*Asclepiadaceae*	AgNO_3_	Ag NPs 4–25 nm	showed strong antibacterial and antifungal activity against some pathogens of bacteria and fungi	[124]
*Hevea brasiliensis*/rubber latex	*Euphorbiaceae*	AgNO_3_	Ag NPs 2–100 nm	not included	[122]
*Citrus aurantium*/oil	*Rutaceae*	Chitosan and glacial acetic acid	*Citrus aurantium* essential oil-loaded chitosan nanoparticles (CAEO-CS NPs) 20–60 nm	led to maintenance of antioxidant properties and hence postharvest quality of button mushroom for longer period of time	[131]
Pine (*Pinus* spp.)/pollen	*Pinaceae*	AgNO_3_	Ag NPs 12 nm	showed significant inhibitory effects against pathogenic fungal growth	[126]
*Phoenix dactylifera*/root extracts	*Palmae*	AgNO_3_	Ag NPs 15–40 nm	exhibited multifunctional properties against human cancer and infectious diseases	[127]
*Acacia rigidula*/stems and roots	*Mimosaceae*	AgNO_3_	Ag NPs 6–66 nm	showed inhibitory activity against dermatophytes	[128]
Screw pine *(Pandanus tectorius)*/leaf cellulose	*Pandanaceae*	H_2_SO_4_ solution	Cellulose nanocrystals 5–25 nm	not included	[130]
Rubberwood *(Hevea brasiliensis)*/stem fiber and oil palm *(Elaeis guineensis)*/fruit pulp	*Euphorbiaceae* and *Palmae*	Chemo-mechanical processes	Cellulose nanofibers 10–90 nm for *H. brasiliensis* and 5–40 nm for *E. guineensis*	not included	[129]
