# Correction: Siam et al. Forest Tree and Woody Plant-Based Biosynthesis of Nanoparticles and Their Applications. *Nanomaterials* 2025, *15*, 845

**DOI:** 10.3390/nano15191534

**Published:** 2025-10-09

**Authors:** Abubakr M. J. Siam, Rund Abu-Zurayk, Nasreldeen Siam, Rehab M. Abdelkheir, Rida Shibli

**Affiliations:** 1 Department of Horticulture & Crop Science, Faculty of Agriculture, University of Jordan, Amman P.O. Box 11942, Jordan; abmjsiam@gmail.com (A.M.J.S.); r.shibli@ju.edu.jo (R.S.); 2 Department of Forestry & Range Science, Faculty of Environmental Sciences & Natural Resources, University of Al Fashir, El Fasher P.O. Box 125, Sudan; 3 Hamdi Mango Center for Scientific Research, The University of Jordan, Amman P.O. Box 11942, Jordan; 4 Department of Industrial Chemistry, College of Applied & Industrial Science, University of Bahri, Khartoum P.O. Box 1660, Sudan; nasreldeennono11@hotmail.com; 5 Department of Forestry, College of Natural Resources & Environmental Studies, University of Bahri, Khartoum P.O. Box 1660, Sudan; rhab_mhmood@hotmail.com

In the original publication [1], the reference [50], “Fakruddin, M.; Hossain, Z.; Afroz, H. Prospects and applications of nanobiotechnology: A medical perspective. *J. Nanobiotechnol*. **2012**, *10*, 31”, was retracted prior to this publication, and the authors would like to delete it. With this correction, the order of some references has been adjusted accordingly. The authors state that the scientific conclusions are unaffected. This correction was approved by the Academic Editor. The original publication has also been updated.
